# Treatment of Liver Metastases from Midgut Neuroendocrine Tumours: A Systematic Review and Meta-Analysis

**DOI:** 10.3390/jcm8030403

**Published:** 2019-03-22

**Authors:** Enes Kaçmaz, Charlotte M. Heidsma, Marc G. H. Besselink, Koen M. A. Dreijerink, Heinz-Josef Klümpen, Elisabeth J. M. Nieveen van Dijkum, Anton F. Engelsman

**Affiliations:** 1 Department of Surgery, Amsterdam UMC, University of Amsterdam, Meibergdreef 9, 1105 AZ Amsterdam, The Netherlands; e.kacmaz@amc.uva.nl (E.K.); c.m.heidsma@amc.uva.nl (C.M.H.); m.g.besselink@amc.uva.nl (M.G.H.B.); e.j.nieveenvandijkum@amc.uva.nl (E.J.M.N.v.D.); 2 Department of Endocrinology, Amsterdam UMC, Vrije Universiteit Amsterdam, 1081 HV Amsterdam, The Netherlands; k.dreijerink@vumc.nl; 3 Department of Oncology, Amsterdam UMC, University of Amsterdam, 1105 AZ Amsterdam, The Netherlands; h.klumpen@amc.uva.nl; 4 ENETS Center of Excellence, Amsterdam UMC, University of Amsterdam, 1105 AZ Amsterdam, The Netherlands; 5 Cancer Center Amsterdam, 1182 DB Amsterdam, The Netherlands

**Keywords:** small bowel neuroendocrine tumours, pancreatic neuroendocrine tumours, liver metastases, midgut, meta-analysis

## Abstract

Strong evidence comparing different treatment options for liver metastases (LM) arising from gastroenteropancreatic neuroendocrine tumours (GEP-NET) is lacking. The aim of this study was to determine which intervention for LMs from GEP-NETs shows the longest overall survival (OS). A systematic search was performed in MEDLINE, Embase and the Cochrane Library in February 2018. Studies reporting on patients with LMs of any grade of sporadic GEP-NET comparing two intervention groups were included for analysis. Meta-analyses were performed where possible. Eleven studies, with a total of 1108, patients were included; 662 patients had LM from pancreatic NETs (pNET), 164 patients from small-bowel NETs (SB-NET) and 282 patients of unknown origin. Improved 5-year OS was observed for surgery vs. chemotherapy (OR 0.05 95% CI [0.01, 0.21] *p* < 0.0001), for surgery vs. embolization (OR 0.18 95% CI [0.05, 0.61] *p* = 0.006) and for LM resection vs. no LM resection (OR 0.15 95% CI [0.05, 0.42] *p* = 0.0003). This is the largest meta-analysis performed comparing different interventions for LMs from GEP-NETs. Despite the high risk of bias and heterogeneity of data, surgical resection for all tumour grades results in the longest overall survival. Chemotherapy and embolization should be considered as an alternative in case surgery is not feasible.

## 1. Introduction

Gastroenteropancreatic neuroendocrine tumours (GEP-NET) represent a heterogeneous group of tumours arising from neuroendocrine cells of the gastro-intestinal tract. The annual incidence of GEP-NETs is estimated to be around 2.88 (European standardized rate, ESR) [1]. In specialized centres, liver metastases (LM) are diagnosed in up to 80–90% of patients with small-bowel NETs (SB-NET) and 60–70% of patients with pancreatic neuroendocrine NETs (pNET) [2]. LM is the strongest predictor for poor survival of patients with GEP-NET regardless of the location of the primary tumour with a 5-year overall survival of 13–54 months for patients with untreated LM [3].

Treatment of patients with LM is aimed at local tumour control and symptom relief. Several treatment modalities for NET-LMs exist, and include resection or debulking of the metastases, radiofrequency ablation (RFA), tumour embolization and pharmacological treatment. Pharmacologic interventions include somatostatin analogues (SSA), targeted therapy, peptide receptor radionuclide therapy (PRRT), chemotherapy and immunotherapy. SSAs reduce hormone associated symptoms in patients, while lengthening progression free survival (PFS) [4,5,6]. The phase 3 NETTER-trial showed improvement in PFS when treating patients with 177-Lu-Dotatate (PRRT) and octreotide with long acting release (LAR) versus octreotide LAR alone in patients with well differentiated metastatic midgut NETs [7]. The protein kinase inhibitor everolimus and sunitinib also increase PFS in patients with advanced NETs [8,9,10]. Hepatic artery embolization (HAE) prolongs survival, whilst being safe and feasible [11]. Current ENETS guidelines state that SSA, octreotide and lanreotide are equally effective in both symptom control and antiproliferative effect [12].

A systematic review published in 2008 by Gurusamy et al. aimed to compare liver resection to other treatment modalities in patients with LMs from GEP-NETs, but were unable to conduct an analysis due to a lack of relevant articles at that time [13]. In the past decade, multiple cohort studies were published. The aim of this systematic review is to determine which treatment modality leads to highest overall survival in patients with LM from GEP-NETs.

## 2. Methods

### 2.1. Search Strategy

A systematic search was performed in MEDLINE (PubMed), Embase (Ovid) and the Cochrane Library on 1 February 2018 (Supplementary Material S1). The search strategy is presented in Supplementary Material S1 and included both keywords and MeSH terms: ‘neuroendocrine tumours’, ‘midgut’, ‘liver metastasis’, ‘pancreatic neoplasms’, ‘duodenal neoplasms’, ‘ileal neoplasms’, ‘jejunal neoplasms’, ‘somatostatin’, ‘interferons’, ‘molecular targeted therapy’, ‘chemotherapy’, ‘surgery’, ‘surgical oncology’, and ‘catheter ablation’. No publication date restriction was used. Studies published in any language other than English were excluded. This study was registered in PROSPERO with the following registration number: CRD42018104328.

### 2.2. In- and Exclusion Criteria

All randomized controlled trials, cross-sectional, cohort studies and case-series reporting on treatment of GEP-NET related LM with at least 5 patients in a minimum of two compared intervention groups were eligible for inclusion. All grades of GEP-NETs were included. Patients with mixed neuroendocrine or non-neuroendocrine neoplasms (MINEN/MENEN) were excluded. No age limit was applied.

### 2.3. Study Selection

All studies identified by the search were screened for eligibility by two independent authors (AE, EK) using Rayyan software (Qatar Computing Research Institute, Doha, Qatar) [14]. After selection based on title and abstract, full texts were analysed for further in- or exclusion. Any conflicts arising from the selection were resolved by consensus. The 5-year overall survival or 5-year disease specific survival after intervention had to be stated in the study, or the data to calculate this had to be available. No strict definition of a curative or palliative resection had to be met. Patients with LM from pancreatic, duodenal, jejunal or ileal NETs were included. In case of publications with overlapping patient cohorts, the study with the largest cohort size was included for analysis.

### 2.4. Data Extraction

The following characteristics were extracted: patient characteristics, primary tumour location (pancreas or small bowel), type of therapy for LM, resection of the primary tumour, LM status (resectable/unresectable), uni- or bilobar metastases, extrahepatic disease, WHO (World Health Organization) 2010 grade and follow-up period. The primary outcome was 5-year overall survival. Secondary outcomes included disease free survival (DFS), progression free survival (PFS) and post-operative complications. Subgroups for analysis were defined as resection of primary tumour versus no resection at all, LM resection versus no resection at all, any resection versus chemotherapy, any resection versus embolization and any resection versus LTx (liver transplantation). ‘No resection at all’ was defined as no LM nor primary resection, ‘any resection’ was defined as a primary with or without LM resection.

### 2.5. Statistical Analysis

For the meta-analysis, outcome data stratified by subgroups were pooled using Review Manager 5.3 (The Nordic Cochrane Centre, The Cochrane Collaboration, Denmark, Copenhagen) and presented in a forest plot. Heterogeneity was assessed by calculating the *I*^2^ index. An *I*^2^ < 25% was considered as low and a fixed effects model was used for the meta-analysis using and the Mantel–Haenszel method [15]. An *I*^2^ between 25–75% was considered as intermediate and consequently a random effects model was used for the meta-analysis. An *I*^2^ > 75% was considered substantial and no meta-analysis was performed. Funnel plots were made to assess publication bias.

### 2.6. Risk of Bias Assessment

The ROBINS-I (Risk of Bias in Non-randomized Studies—of Intervention) tool was used to assess risk of bias for the included studies [16].

## 3. Results

### 3.1. Description of Studies

**Figure 1:**
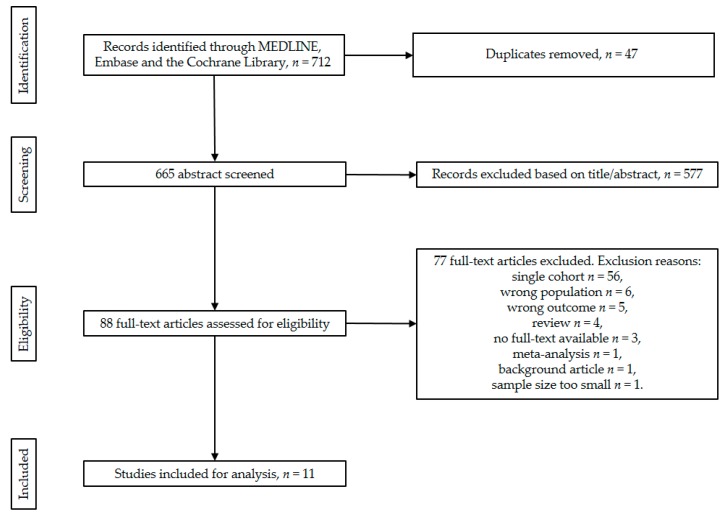
PRISMA flow chart of the study screening and selection process.

A total of 712 studies were identified through the electronic search in MEDLINE (PubMed), Embase and the Cochrane Library. After the screening and selection process, 11 studies fulfilled the inclusion criteria (Figure 1) [17,18,19,20,21,22,23,24,25,26,27]. Characteristics of the included studies are presented in Table 1. There were no randomized controlled trials found. The 11 included studies represent a total of 1108 patients, of which 662 patients had pNETs, 164 patients had SB-NETs and 282 patients had a tumour originating from lungs (*n* = 26), ovaries (*n* = 1) and unknown primary locations (*n* = 102) (Table 2). Out of all included studies, five intervention groups were composed: primary tumour resection versus no resection at all, LM resection versus no resection at all, any resection versus chemotherapy, any resection versus embolization and any resection versus LTx.

### 3.2. Resection of Primary Tumour versus No Resection at All

**Figure 2:**
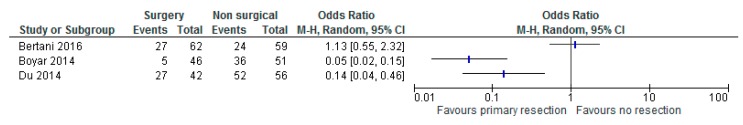
Forest plot for overall survival (OS) after resection of primary tumour versus no resection at all.

This intervention group compares primary resection versus no primary resection with LM presence in both groups. Three studies reported outcomes on resection of primary tumour (*n* = 150) versus no resection of primary tumour (*n* = 166) with a total number of 365 patients [17,18,24]). High statistical heterogeneity based on an I^2^ of 92% withheld us from conducting a meta-analysis with these studies (Figure 2).

### 3.3. LM Resection versus No Resection at All

**Figure 3:**
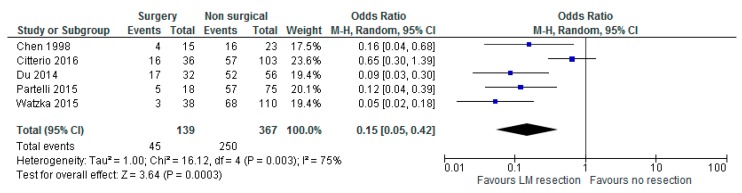
Forest plot for overall survival (OS) after liver metastases (LM) resection versus no resection at all.

Five studies reported outcomes on resection of LM (*n* = 139) versus no resection (*n* = 367) with a total number of 506 patients [20,21,24,26,27]). Chen et al. reported a median DFS of 21 months after LM resection [20]. Partelli et al. reported a median DFS of 42, 27 and 15 months after curative, palliative and no surgery, respectively [26]. Statistical heterogeneity amounted to 75% thus a meta-analysis was performed. The meta-analysis resulted in a statistically significant benefit in 5-year OS (overall survival) in favour of LM resection versus no resection at all (OR 0.15 with 95% CI 0.05–0.42, *p* = 0.0003, Figure 3).

### 3.4. Any Surgery versus Chemotherapy

**Figure 4:**
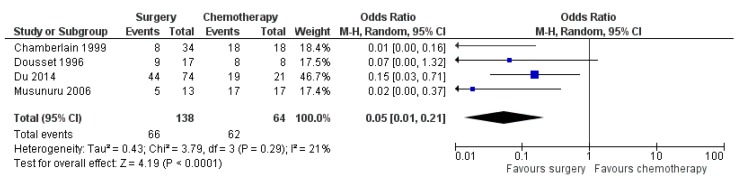
Forest plot for overall survival (OS) after any surgery versus chemotherapy.

Four studies reported outcomes on surgery (*n* = 138) versus chemotherapy (*n* = 64) with a total number of 202 patients (19, 23–25). Additional therapy was provided for two out of 32 patients in the surgery group with either TACE or RFA in the study by Du et al. [24]. Statistical heterogeneity amounted to 21%, thus a meta-analysis was performed. The meta-analysis resulted in a statistically significant 5-year OS in favour of any surgery versus chemotherapy (OR 0.05 with 95% CI 0.01–0.21, *p* < 0.0001, Figure 4).

### 3.5. Any Surgery versus Embolization

**Figure 5:**
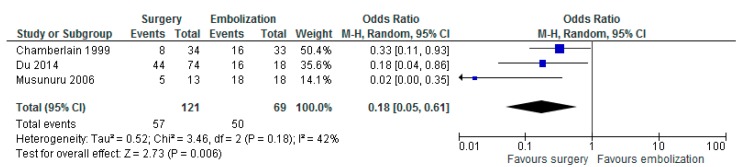
Forest plot for overall survival (OS) after any surgery versus embolization.

Three studies reported outcomes on surgery (*n* = 121) versus embolization (*n* = 69) with a total number of 190 patients [19,24,25]. Statistical heterogeneity amounted to 42%, thus a meta-analysis was performed. The meta-analysis resulted in a statistically significant OS in favour of any surgery versus embolization (OR 0.18 with 95% CI 0.05–0.61, *p* = 0.006, Figure 5).

### 3.6. Any Surgery versus LTx

**Figure 6:**
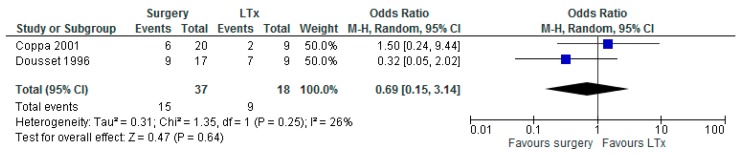
Forest plot for overall survival (OS) after any surgery versus liver transplantation (LTx).

Two studies reported outcomes on surgery (*n* = 37) versus LTx (*n* = 18) with a total number of 55 patients [22,23]. Studies used strict criteria for patients to be eligible for LTx. Statistical heterogeneity amounted to 26%, thus a meta-analysis was performed. The meta-analysis showed no difference in OS regarding any surgery versus LTx (OR 0.69 with 95% CI 0.15–3.14, *p* = 0.64, Figure 6). Coppa et al. reported a median DFS of 24 months after hepatic resection [22]. Dousset et al. reported a median DFS of 17 months after curative and palliative surgery and 19.5 months after LTx [23].

### 3.7. Risk of Bias

In accordance with the ROBINS-I guidelines, the overall risk of bias was scored as critical for all studies (Table 3), the reason being that all studies scored a critical risk of bias in the ‘bias due to confounding’ domain due to the lack of randomized controlled trials. The funnel plots show that, as expected, some publication bias is present in the included study (Supplementary Material S2).

## 4. Discussion

Surgical resection of LM with curative intent is the current standard of care [2]. The aim of this treatment strategy is to prolong OS and maintain quality of life. This systematic review presents the first meta-analysis, involving 11 cohort studies and 1108 patients, comparing surgery with other treatment modalities for GEP-NET related LM. The meta-analysis showed a significantly improved 5-year OS after LM resection versus no resection at all, after any surgery versus chemotherapy and after any surgery versus embolization. No significant benefit of any surgery as compared to LTx was observed.

Although our results are heterogeneous, they are supported by a recent study from Yu et al. [28]. In this study, a systematic review and meta-analysis were performed comparing liver resection with non-liver resection treatments for patients with LM from all grades of pNET. The meta-analysis resulted in a median 5-year OS of 68% in the liver resection group, and 27% in the non-liver resection group. Survival outcomes reached statistical significance for 5-year OS with an OR of 5.30 (95% CI [3.24, 8.67] *p* < 0.001), in favour of liver resection.

A number of studies in this systemic review also reported an improved DFS in favour of surgery versus other treatments. However, because of the limited data reported, no meta-analysis could be performed [20,22,23,25]. Data regarding complications was limited, only two studies reported complications due to hepatic surgery [26,27]. Different from an earlier published Cochrane review, cohort studies were considered for inclusion, which enabled the meta-analysis [13]. Although this study was not able to conduct a meta-analysis comparing primary tumour resection to no primary resection, a trend towards a beneficial effect of primary tumour resection is observed and supported by other studies [29,30]. In addition, performing LTx remains a topic of debate due to the small number of patients reported in the literature [31].

This review also included patients with metastases of WHO grade 3 SB-NETs. These patients showed an improved 5-year OS after resection of the LMs. This supports the ENETS 2012 guideline regarding an indication for resection of LM in WHO grade 3 NETs whenever possible, assessed per individual case [2]. We agree with the ENETS 2012 guideline; however, we also propose that the presence of extrahepatic metastases should not be an exclusion criterion, but that resection should be, again, considered per individual case [2]. Our data supports the updated ENETS 2016 guideline, stating that ablative therapies should be considered when surgery is contraindicated in LM from grade 1 and grade 2 NETs (Figure 5) [12].

This systematic review and meta-analysis have a number of limitations, mainly due to the rarity of the disease and limited conducted interventional studies, with a lack of randomized controlled trials (RCT). This resulted in inclusion of 11 retrospective cohort studies, resulting in a low level of evidence (level C) [32]. As a consequence, drawing conclusions is challenging due to a high risk of selection bias, but hypothesis generating remains possible. Moreover, the included studies have small cohort sizes on subgroup level, interventions were performed on different tumour grades and the studies used a variety of types of individual interventional approaches. It is also unfortunate that no quality-of-life data were reported in the included studies. Because our analyses are based only on published data, there is also a risk of publication bias. Despite the obvious drawbacks of this study, it is at present the best available evidence.

Even though a systematic approach was used in this study, the data is of limited quality and the question of which intervention yields the most benefit for OS in patients with LM from pNET/SB-NET remains unanswered. Randomized trials would generate evidence of great quality, but the execution of such a study is challenging (due to the long follow-up time needed and financial burden, among other things). Therefore, further prospective multi-centre research should address this question, for example by collaboration of multiple ENETS Centers of Excellence. Dousset et al. and Partelli et al. also report underestimation of liver disease by preoperative imaging studies, indicating room for improvement [23,26].

Watzka and colleagues reported on the largest included cohort of LM from GEP-NET [27].

In multivariate analysis, occurrence of synchronous or metachronous LM, hormonal activity and the site of the primary tumour were not independent significant prognostic factors, whereas tumour grade and resection margin status were. These prognostics factors should be taken into account when designing new studies.

Currently, a randomized trial is being conducted, comparing the resection of primary tumours vs. no resection of primary tumours in asymptomatic patients with unresectable LM from SB-NET (NCT03442959). However, survival analyses are not expected soon.

Surgical resection of LMs from all grades GEP-NETs should be considered if possible, and chemotherapy and embolization should be considered as an alternative in case surgery is not feasible. We therefore advocate that all patients with LM from pNET/SB-NET should be discussed in referral centers with specialized multidisciplinary meetings for NETs, preferably in ENETS Centers of Excellence.

## Figures and Tables

**Table 1:** Characteristics of included studies.

Author	Country	Design	No. Patients (*n*)	Inclusion Criteria Per Study	Exclusion Criteria Per Study	Intervention Groups	Control Group
Watzka et al. [27]	DE	Retrospective	204	Patients with LM of NEN.	N/A	Radical LM resection (*n* = 38)	No resection at all (*n* = 110)
Partelli et al. [26]	IT	Retrospective	166	Patients with synchronous LM from sporadic pNET.	Patients with extra-abdominal disease as well as those with peritoneal carcinomatosis and those with an inherited syndrome.	Radical LM resection + primary resection (*n* = 18)	No resection at all (*n* = 75) (SSA; PRRT; chemotherapy; everolimus or sunitinib)
Citterio et al. [21]	IT	Retrospective	139	≤20 mitoses/10 high power field (HPF) and Ki-67 labelling index ≤ 20% at either the primary or metastatic sites; Hormone-secreting status associated with a distinct clinical syndrome (functioning NETs); Performance status (PS) 0–1 at presentation, according to the ECOG ^§^	N/A	LM resection (*n* = 36) (32 were after primary resection)	No resection at all (*n* = 103) (SSA *n* = 95, SSA + chemo *n* = 30, SSA + everolimus *n* = 14, TACE or RFA + systemic and/or surgical treatment * *n* = 25)
Du et al. [24]	CN	Retrospective	130	LM from NET.	N/A	Radical resection of primary tumour (*n* = 42)	No resection at all (*n* = 56) (TACE (16/18 also received an RFA) *n* = 18, systemic chemotherapy *n* = 9, SSA *n* = 12, no treatment *n* = 17)
						LM + primary resection (R0) *n* = 26, LM resection (R0) *n* = 6	
						Primary + LM resection *n* = 26, primary resection *n* = 42, LM resection *n* = 6	Chemotherapy (*n* = 21) chemotherapy (fluorouracil and/or epirubicin and/or doxorubicin and/or etoposide and/or cisplatin, etc.) *n* = 9, SSA *n* = 12)
							TACE (*n* = 18) (16 also received a RFA)
Bertani et al. [17]	IT	Retrospective	121	Patients with synchronous and unresectable pNET LM.	N/A	Resection of primary tumour (*n* = 62) (*n* = 59 also received PRRT)	No resection at all (*n* = 59) (PRRT *n* = 55, SSA *n* = 29)
Boyar et al. [18]	NO	Retrospective	114	Patients with (WHO 2010) grade 1 and grade 2 tumours.	N/A	Resection of primary tumour with curative intent (*n* = 46)	No resection at all (*n* = 51) (streptozotocin + 5-fluorouracil/doxorubicin; SSA; IFN; embolization; PRRT; M-tor inhibitor)
Chamberlain et al. [19]	US	Retrospective	85	Patients treated for hepatic NET metastases.	The absence of identifiable liver disease, pathologic review at MSKCC revealing a non-NET or high-grade NET, or a patient decision to seek care elsewhere.	Segmentectomy or enucleation *n* = 12, lobectomy *n* = 3, extended resection *n* = 19 ^‡^	Chemotherapy (*n* = 18) (streptozocin+ 5-FU; streptozocin + doxorubicin; 5-FU + leucovorin or cisplatin + etoposide)
							HAE, with polyvinyl alcohol particles (*n* = 33)
Musunuru et al. [25]	US	Retrospective	48	Patients with liver-only metastatic neuroendocrine tumours.	N/A	Anatomical liver resection *n* = 6, ablation *n* = 4, resection and ablation *n* = 3	Chemotherapy (*n* = 17) (observation, octreotide, and/or systemic chemotherapy)
							Embolization (*n* = 18)
Chen et al. [20]	US	Retrospective	38	Patients treated for hepatic NET metastases.	Patients with evidence of extrahepatic disease or unresected known primary tumour.	LM resection (*n* = 15) (12 were combined with primary resection)	No resection at all (*n* = 23) (chemoembolization *n* = 5, chemotherapy and radiation *n* = 6, chemotherapy only *n* = 3, radiation only *n* = 2, no therapy *n* = 7)
Dousset et al. [23]	FR	Retrospective	34	Patients with metastatic endocrine tumours with bilobar metastases.	N/A	Curative intent resection *n* = 12 Palliative intent *n* = 5 †	Chemotherapy (*n* = 8) (streptozotocin + fluorouracil *n* = 4, chemoembolization *n* = 4)
							LTx (*n* = 9)
Coppa et al. [22]	IT	Retrospective	29	LM from NET, confirmed histological diagnosis.	Non-carcinoid primary tumours, tumours with systemic venous drainage.	Hepatic resection with curative intent (*n* = 20)	LTx (*n* = 9)

IFN: interferon, IT: Italy, NO: Norway, US: United States, FR: France, 5-FU: 5-fluoro-uracil, CN: China, DE: Germany, pNET: pancreatic neuroendocrine tumours, NET: neuroendocrine tumours, LM: liver metastases, LTx: liver transplantation, NEN: neuroendocrine neoplasms, N/A: not available, PRRT: peptide receptor radionuclide therapy, RFA: radiofrequency ablation, SSA: somatostatin analogues; TACE: transarterial chemoembolization; * these interventions were also received by patients in the LM resection group; † *n* = 4 received additional chemotherapy and *n* = 4 chemoembolization; ^‡^ 28/34 with a curative intent; ^§^ Eastern Cooperative Oncology Group; ^¶^ Memorial Sloan Kettering Cancer Center.

**Table 2:** Patient characteristics of included studies.

Study	No. Patients (*n*)	Sex (n, %)		Age (Years)	Primary Tumour Location			LM Size in cm (Median, Range)	Non-Functional NETs (*n*, %)	Resection of Primary Tumour (*n*, %)	Resectable/Unresectable LM	Uni-/Bilobar Metastases	Extrahepatic Disease (*n*, %)	WHO 2010 Grade
		Male	Female		Pancreas (*n*, %)	Small Bowel (*n*, %)	Other/Unknown (*n*, %)							
Watzka et al. [27]	204	111 (54)	93 (46)	58 ± 15 (60) *	58 (28)	73 (36)	73 (36)	N/A	123 (60)	165 (81)	Mixed	N/A	N/A	All
Partelli et al. [26]	166	92 (55)	74 (45)	N/A ^‡^	166	0	0	LM resection 0.8 cm (0.3–1.7 cm); no resection at all 3.4 cm (1–7 cm) †	152 (92)	91 (55)	Resectable	Both	N/A	All
Citterio et al. [21]	139	67 (48)	72 (52)	56 (51–55) ^†^	36 (26)	66 (47)	37 (27)	N/A	0	93 (67)	Mixed	N/A	N/A	1–2
Du et al. [24]	130	69 (53)	61 (47)	49.0 ± 12.1 (N/A) *	85 (65)	7 [5]	38 (30)	Mean 4.1 cm (range 3–15 cm)	100 (77)	68 (52)	Mixed	N/A	N/A	All
Bertani et al. [17]	121	66 (55)	58 (45)	54.6 ± 12.6 (54.5) *	121 (100)	0	0	N/A	29 (24)	63 (52)	Unresectable	N/A	28 (23)	All
Boyar et al. [18]	114	61 (54)	83 (46)	57 (32–83) ^†^	111 (97)	0	3 [3]	N/A	89 (78)	46 (40)	Mixed	N/A	51 (45)	1–2
Chamberlain et al. [19]	85	37 (44)	48 (56)	52 (20–79) ^†^	42 (49)	0	43 (51)	N/A	49 (58)	36 (42)	Mixed	Both	45 (53)	1–2
Musunuru et al. [25]	48	30 (63)	18 (37)	N/A	15 (31)	0	33 (69)	Embolization 8.9 ± 6.1 cm; chemotherapy 3.7 ± 2.9 cm; any resection 4.5 ± 2.3 cm *	N/A	12 (25)	Unclear	Both	0	N/A
Chen et al. [20]	38	24 (63)	14 (37)	N/A ^‡^	11 (29)	9 (24)	18 (47)	N/A	9 (24)	12 (32)	Mixed	Bilobar	0	N/A
Dousset et al. [23]	34	18 (53)	17 (47)	49.5 (29–76) ^†^	17 (50)	9 (26)	8 (24)	N/A	5 (15)	21 (62)	Mixed	Bilobar	0	N/A
Coppa 2001 et al. [22]	29	13 (45)	16 (55)	N/A ^‡^	0	0	29^§^	N/A	N/A	11 (38)	Mixed	N/A	0	N/A

* mean ± SD (median); ^†^ median (range); ^‡^ Age was reported for each subgroup separately; ^§^ 21 have a pancreatic or ileal origin, whilst 8 originated in the lung or rectum; N/A: not available.

**Table 3:** Risk of bias in included studies scored with the ROBINS-I tool.

	Bias Due to Confounding	Bias in Selection of Participants into the Study	Bias in Classification of Interventions	Bias Due to Deviations from Intended Interventions	Bias Due to Missing Data	Bias in Measurement of Outcomes	Bias in Selection of the Reported Result	Overall Bias
Chamberlain et al. [19]	-	+/-	+	+	+/-	+/-	+/-	-
Coppa et al. [22]	-	+/-	+	+	+/-	+/-	+/-	-
Du et al. [24]	-	+/-	+	+	+/-	+/-	+/-	-
Musunuru et al. [25]	-	+/-	+	+	+/-	+/-	+/-	-
Boyar et al. [18]	-	+/-	+/-	+	+/-	+/-	+/-	-
Bertani et al. [17]	-	+/-	+	+/-	+/-	+/-	+/-	-
Chen et al. [20]	-	+/-	+	+/-	+/-	+/-	+/-	-
Citterio et al. [21]	-	+/-	+	+/-	+/-	+/-	+/-	-
Partelli et al. [26]	-	+/-	+	+/-	+/-	+/-	+/-	-
Watzka et al. [27]	-	+/-	+	+/-	+/-	+/-	+/-	-
Dousset et al. [23]	-	+/-	+/-	+/-	+/-	+/-	+/-	-

+: low (green); +/-: moderate (yellow); -: critical (red).

## Supplementary Materials

The following are available online at https://www.mdpi.com/2077-0383/8/3/403/s1, Supplementary Material S1: Search strategy, Supplementary Material S2: Funnel plots.
